# Does a child’s language ability affect the correspondence between parent and teacher ratings of ADHD symptoms?

**DOI:** 10.1186/s12888-017-1300-8

**Published:** 2017-04-05

**Authors:** Debbie Gooch, Harriet Maydew, Claire Sears, Courtenay Frazier Norbury

**Affiliations:** 1 Department of Psychology, Royal Holloway, University of London, Egham, TW20 0EX UK; 2 Division of Psychology & Language Sciences, University College London, Chandler House, 2 Wakefield Street, London, WC1N 1PF UK

**Keywords:** Language, Attention-Deficit/Hyperactivity Disorder, Rating scales, Inter-rater reliability

## Abstract

**Background:**

Rating scales are often used to identify children with potential Attention-Deficit/Hyperactivity Disorder (ADHD), yet there are frequently discrepancies between informants which may be moderated by child characteristics. The current study asked whether correspondence between parent and teacher ratings on the Strengths and Weakness of ADHD symptoms and Normal behaviour scale (SWAN) varied systematically with child language ability.

**Method:**

Parent and teacher SWAN questionnaires were returned for 200 children (aged 61–81 months); 106 had low language ability (LL) and 94 had typically developing language (TL). After exploring informant correspondence (using Pearson correlation) and the discrepancy between raters, we report inter-class correlation coefficients, to assess inter-rater reliability, and Cohen’s kappa, to assess agreement regarding possible ADHD caseness.

**Results:**

Correlations between informant ratings on the SWAN were moderate. Children with LL were rated as having increased inattention and hyperactivity relative to children with TL; teachers, however, rated children with LL as having more inattention than parents. Inter-rater reliability of the SWAN was good and there were no systematic differences between the LL and TL groups. Case agreement between parent and teachers was fair; this varied by language group with poorer case agreement for children with LL.

**Conclusion:**

Children’s language abilities affect the discrepancy between informant ratings of ADHD symptomatology and the agreement between parents and teachers regarding potential ADHD caseness. The assessment of children’s core language ability would be a beneficial addition to the ADHD diagnostic process.

**Electronic supplementary material:**

The online version of this article (doi:10.1186/s12888-017-1300-8) contains supplementary material, which is available to authorized users.

## Background

Attention-Deficit/Hyperactivity Disorder (ADHD) is a developmental disorder characterised by persistent and pervasive symptoms of inattention, hyperactivity and impulsivity [1]. Diagnosis of ADHD necessitates that symptoms are present in at least two settings and rating scales are often used as the first step in the diagnostic process to obtain feedback from multiple informants, usually parents and teachers [2]. However, weak correlations and discrepancies between parent and teacher ratings have been consistently reported [3–6].

Parents are able to witness their child’s development through the years in different situations and can therefore provide a holistic view of their behaviour [7]. Teachers, on the other hand, interact with many children of the same age and hence may be able to identify atypical behaviours more easily [8]. Thus parents and teachers may have different benchmarks not only because they are observing a child’s behaviour in different situations, but also because they have different motivations for providing certain ratings or different expectations of what constitutes normal/abnormal behaviour [9].

As there is no single measure with which to assess the extent to which a child is experiencing ADHD symptoms, it is challenging to assess the validity of individual informant ratings [5]. Nevertheless, informant discrepancy means that relying on one informant or even integrating information from multiple informants could lead to different conclusions regarding the correlates of or risk factors for disorder [10]. Moreover, informant discrepancy could affect estimates of disorder prevalence and associated comorbidity [5].

Factors that might influence correspondence between informants include child characteristics such as age, gender, ethnicity, behavioural profile, and perceived functional impact [11–13]. Although informant discrepancies have been found to be moderated by some of these factors, findings are inconsistent [5]. The current study examines a factor which to date has been under-researched; the effect of the child’s language ability on informant ratings of ADHD symptomology.

### Why is language ability an important factor?

Language impairment frequently co-occurs with ADHD, with co-morbidity rates of 30–50% [14, 15]. However, some have argued that these elevated rates reflect overlapping symptoms that arise from distinct causal pathways [16]. For instance, many ADHD rating scales contain items tapping academic problems [16] which could arise from either core language deficits or as sequelae of attention deficits (e.g. does not seem to listen to what is being said; does not follow through on instructions/finish school work). Teachers who observe a child’s behaviour within a classroom setting maybe more strongly influenced by a child’s academic attainment than parents and thus more likely to endorse items which reflect language/academic problems when rating symptoms of ADHD in children with language difficulties. Thus level of language competence may particularly influence teacher ratings of ADHD and accentuate discrepancies between parent and teacher ratings.

Deficient language ability may also differentially affect ratings of ADHD symptoms through the informant’s susceptibility to negative halo effects, a cognitive bias in which an informant’s overall impression of an individual influences ratings of specific characteristics. Halo effects have been demonstrated in teacher ratings of ADHD and Oppositional Defiant Disorder (ODD) [17] with teachers rating children with ODD as having high levels of inattention and hyperactivity even when these behaviours were not evident. These effects were less evident in a similar study looking at parent ratings of ADHD and ODD symptoms [18] suggesting that parents may be less susceptible to halo effects. Similarly, children with language difficulties may be perceived to be more inattentive/hyperactive, though these perceptions may be tempered by the language demands of the environment (e.g. school vs. home).

To date there is little research investigating whether correspondence among parent and teacher ratings of ADHD symptoms varies systematically as a function of children’s language competence. Although there is little evidence for an association between children’s verbal IQ and the discrepancy between parent and teacher ratings of ADHD symptoms [19], when parents and teachers agree on a child’s language status (impaired vs. no impairment), their agreement regarding behavioural-emotional problems is greater [20]. Furthermore, teachers report higher levels of conduct problems and hyperactivity for children with lower cognitive/verbal ability than parents [10]. Similarly, preschool children with advanced pre-academic skills, including language ability, received lower ratings of attention problems from their teachers compared to their mothers, while children with poorer pre-academic skills were more likely to be rated by teachers as having attention deficits [21]. Together these findings suggest that children’s language ability may differentially affect parent and teacher ratings of ADHD symptoms; with teachers being more sensitive to a child’s language ability when rating their behaviour within a classroom setting [21].

## Methods

### Aims

The current study aimed to compare parent and teacher ratings of behaviour on a relatively new dimensional measure of ADHD symptomatology, the Strengths and Weaknesses of ADHD symptoms and Normal behaviour (SWAN) rating scale [22]. A novel question concerns the influence of child language skill on the magnitude of respondent discrepancies on the SWAN, as well as the extent to which child language moderates two distinct metrics of agreement. First, we examine the linear correlation between informant ratings, as is common in the literature regarding assessment of childhood psychopathology [8, 10, 23–25]. Although reporting linear correlations provides information regarding the strength of the association between ratings from different respondents, they do not capture agreement between raters and thus systematic and consistent differences between raters can be overlooked [26]. We therefore also report two metrics of inter-rater agreement in relation to language status: (1) inter-class correlation co-efficients (ICCs), which provide an index of inter-rater reliability and the extent to which there are systematic differences in informant agreement [26] and (2) Cohen’s kappa, which assesses parent-teacher agreement regarding significant symptoms of ADHD (i.e. “caseness”). We hypothesised that where discrepancies exist between parent and teacher ratings, child language will have a greater impact on teacher ratings of ADHD symptomatology given the limitations that language ability places on classroom performance.

### Study population

Participants were recruited from the Surrey Communication and Language in Education Study (SCALES), a longitudinal population study of risk for language impairment at school entry [27, 28]. In stage 1, teachers in state-maintained mainstream reception classrooms (age 4–5 years: equivalent to US Kindergarten) in Surrey, England completed online assessments of language using the Children’s Communication Checklist-Short, (CCC-S:[29]), behaviour (the Strengths and Difficulties Questionnaire, SDQ:[30]), and early educational attainment using the Early Years Foundation Stage Profile, (EYFSP:[31]) for 6459 monolingual children aged between 57 and 70 months.

The CCC-S is a short form of the Children’s Communication Checklist-2 [32] and was used to identify children with low levels of language proficiency at school entry. It contains 13 items, tapping children’s use of vocabulary, grammar, pragmatics and speech, that best discriminated cases and controls in a validation study [33], with high degrees of internal consistency (Cronbach’s α = .95, SCALES sample). Teachers rated the frequency with which a range of language behaviours occur in everyday contexts on a 4-point scale, with higher scores reflecting greater impairment. Children reported as having ‘no phrase speech’ and those with CCC-S scores roughly equivalent to 1SD above expected range for sex and age group (autumn, spring or summer born) were classified as having low language ability (LL) [28]. The remaining children were classified as having typical language ability (TL).

In stage 2, 636 monolingual children (aged 61-82 months) were selected for in-depth assessment through stratified (by age-group and sex) random sampling, with a higher sampling fraction for those with LL. As part of a larger battery, 529 (83% of invited children) children completed six measures of language competence assessing vocabulary (Expressive/Receptive One Word Picture Vocabulary Tests [34]); grammar (Test for Reception of Grammar-2 [35] and School-Age Sentence Imitation Test-E32 [36]); and narrative (recall and comprehension; Assessment of Comprehension and Expression 6-11 [37]). Raw scores were adjusted for age and standardised using the full weighted sample of children, yielding a total language composite. In addition, parents and teachers were asked to complete a set of questionnaires including the SWAN. Completed SWAN questionnaires were returned from 299/529 (57%) parents and 346/529 (65%) teachers (see recruitment flow diagram in Additional file 1: Figure S1).

### Consent procedures

Opt-out consent was adopted for stage 1 as data could be provided anonymously to the research team; 20 families opted-out. At stage 2 written, informed consent was obtained from the parents or legal guardians of all participants and verbal assent was obtained from child participants. The consent procedures and study protocol were developed in consultation with Surrey County Council and approved by the Research Ethics Committee at Royal Holloway, University of London.

### Participants

Data presented here are from the 200/529 (38%) monolingual children for whom both parent and teacher SWAN questionnaires were returned in Year 1 (age 5-6 years: equivalent to US Grade 1) (Table 1).^1^ The sample consisted of 102 females and 98 males; 106 (53%) children were classified as having LL (17 of which were reported as having ‘no phrase speech’ at screen) and 94 (47%) as having TL. On average children with LL obtained z-scores on the CCC-S of nearly 1.5 SD below the population average and significantly lower total language composite scores than children with TL. Thus, children rated by their teacher as having LL in Reception also performed poorly on objective measures of language competence one year later.

**Table 1:** Demographics of the study sample (means (SD))

	Typical language	Low language	F Values	*p* Values
N	94	106		
Age	71.52 (4.84)	72.20 (4.62)	F(1199) = 1.02	*p* = .31
% male	45.74	51.89	Chi2(1) = 0.75	*p* = .39
CCC-S (raw score/max 39)	5.34 (5.65)	24.74 (8.19)	F(1199) = 386.54	*p* < .001
CCC-S (z-score)	−.40 (.93)	1.48 (.38)	F(1199) = 335.33	*p* < .001
Language composite (z-score)^1^	.36 (.97)	−.81 (.97)	F(1191) = 69.99	*P* < .001

*Note*: CCC-S z-scores are raw scores adjusted for age and sex and standardised on the whole sample participating at Stage 1; they have a mean of 0 and SD of 1 with higher scores indicating more impairment; ^1^8 children with LL were unable to complete the 6 core language tests

### Strengths and weaknesses of ADHD symptoms and normal behaviour (SWAN)

The SWAN [22] was completed by parents and teachers at stage 2 to assess symptoms associated with ADHD. This dimensional scale was constructed to overcome issues that characterises many other ADHD rating scales, namely that scores are not normally distributed in the population as items focus on the presence of difficulty (Achenbach, [38]; Barkley & Murphy, [39]; Conners, Sitarenios, Parker, & Epstein, [40]; Swanson et al. [22]). Thus the SWAN captures variance at both the positive and negative ends of the ADHD symptom dimension [41]. The SWAN has been reported to have high internal consistency, moderate test-retest reliability and adequate convergent and discriminant validity in preschool children [42]. Although there are studies which report and compare parent and teacher ratings on the SWAN [25, 43] currently there are no published studies specifically exploring the inter-rater reliability (beyond using simple correlations) of the SWAN in English either as a dimensional scale or as a tool to identify possible cases of ADHD.

The SWAN measures the 18 core symptoms of ADHD [1]. Nine items tap inattention (e.g. give close attention to detail and avoid making mistakes) and nine items tap hyperactivity/impulsivity (e.g. awaits turn: stands in line and take turns). Respondents were asked to compare their child’s behaviour to those of their peers using a 7 point Likert scale (1: Far Below Average - 7: Far Above Average) [44]. The maximum score on each SWAN subscale is 63 with lower scores reflecting more inattention/hyperactivity. Cronbach’s alphas in the current sample indicated good internal reliability for the SWAN subscales and total score as completed by parents and teachers (*r*s = .94-.98).

Responses were also coded to identify potential ‘cases’ or children rated as having clinically significant symptoms of ADHD. In line with previous studies, items rated 1 or 2 (far below average and below average respectively) were scored as positive symptoms of ADHD [39, 45]. Consistent with DSM-5 guidelines, children were classified as possible cases of ADHD if six or more symptoms of inattention and/or six or more symptoms of hyperactivity/impulsivity were endorsed. This procedure was repeated for both parent and teacher ratings.

### Data analysis

All analyses were conducted in STATA/IC 12 [46]. After reporting the correlation between parent and teacher ratings on the SWAN, we explored the discrepancy between parent and teacher ratings and the potential moderating role of child language group status. SWAN scores were entered into three repeated measures mixed models, with restricted maximum likelihood estimation. Respondent (parent vs. teacher) was the within subjects factor and language group (LL vs. TL) was the between subjects factor.

Further analyses investigated the agreement between parent and teacher ratings. We assessed inter-rater reliability using intra-class correlation coefficients (ICC) for both the entire sample as well as by language group. ICCs were calculated using random effects models with restricted maximum likelihood estimation. The corresponding ICC reliability coefficients can be interpreted as follows: <.40 are poor, .40-.59 fair, .60-.74 good and .75-1.00 excellent [47]. Finally, Cohen’s kappa was calculated to assess the agreement between parents and teachers regarding identification of ‘caseness’ or clinically significant symptoms of ADHD across the sample and by language group. These can be interpreted as follows: < 0 no agreement, .0–.20 poor, 0.21–0.40 fair, 0.41–0.60 moderate, 0.61–0.80 substantial and 0.81–1 almost perfect agreement [48].

## Results

### The correlation between parent and teacher ratings

**Fig. 1:**
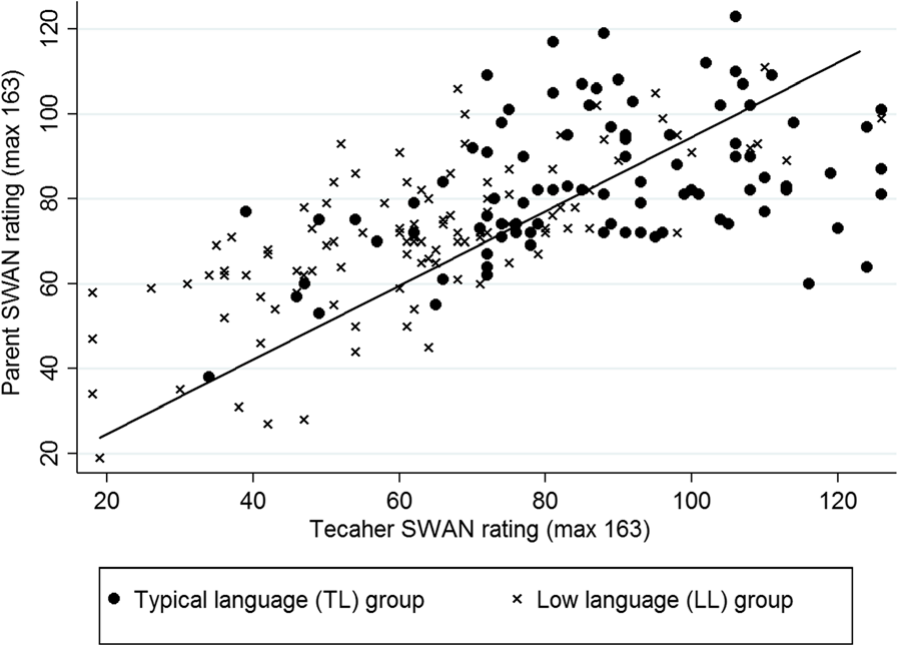
Correlation between parent and teacher ratings on the SWAN total score

Overall the correlations between parent and teacher ratings on the SWAN and its subscales were moderate/strong (SWAN total, *r* = .64; inattention, *r* = .66; hyperactivity, *r* = .55; see Fig. 1 for scatterplot of SWAN total scores).

### Discrepancy between parent and teacher ratings

**Fig. 2:**
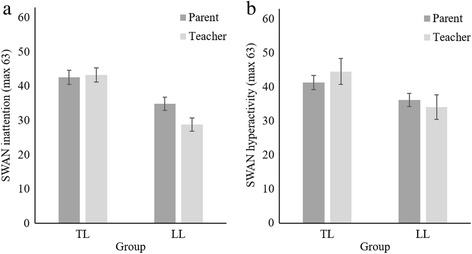
Estimated marginal means of SWAN inattention (**a**) and hyperactivity (**b**) ratings by group. Note: Error bars are 95% CIs; low scores reflect more inattention/hyperactivity

Figure 2 illustrates mean SWAN subscale ratings (with 95% CIs) by respondent for the LL and TL groups (see Additional file 1: Table S2 for means and SDs and Additional file 1: Figure S2 for SWAN total scores by respondent for the two language groups). Teachers generally rated children has having significantly greater inattention compared to parents (z = −3.95, *p* < .001); parent and teacher ratings of hyperactivity, on the other hand, were very similar (z = 0.78, *p* = .44). For both SWAN subscales there were significant effects of language group indicating that children with LL were rated as exhibiting more inattention and hyperactivity than children with TL (inattention: z = −8.90, *p* < .001 and hyperactivity: z = −6.25, *p* < .001).

In addition, there were significant respondent x language group interactions for each of the SWAN subscales (inattention: z = −5.00, *p* < .001 and hyperactivity: z = −3.76, *p* < .001) indicating that the difference between parent and teacher ratings on the inattention subscale was greater for children with LL than for children with TL. In contrast, hyperactivity scores were more discrepant for children with TL, whereas scores between respondents for children with LL were marginally more consistent.

Both parents and teachers rated children with LL as having more inattention than peers (z = −5.47, *p* < 0.001 and z = −10.21, *p* < 0.001 respectively). However, tests of simple effects indicated that for SWAN inattention, teacher ratings for the LL group were significantly worse than parents (z = −6.53, *p* < .001), whereas there was no significant difference between parent and teacher ratings for the TL group (z = .72, *p* = .47).

SWAN hyperactivity ratings further indicated that although both parents and teachers rated the LL group as exhibiting more hyperactivity than the TL group (z = −3.51, *p* < 0.001 and z = −7.30, *p* < 0.001 respectively), teacher ratings of children with LL were significantly worse than parents (z = −2.18, *p* < .05), whereas teacher ratings of children with TL were significantly better than parents (z = 3.12, *p* < .01).

Consideration of CCC-S language scores as a continuous variable yielded comparable findings. Linear correlation between CCC-S and SWAN sub-scales scores were significantly stronger for teachers (*r* = −.66 inattention; −.54 hyperactivity), than for parents (*r* = −.50 inattention; −.37 hyperactivity; z = 2.35 and 2.12 respectively, *p*s < .05).

### Does children’s language ability affect the inter-rater reliability of the SWAN?

Table 2 shows ICCs with 95% confidence intervals for the SWAN and its subscales as an estimate of inter-rater reliability for the whole sample and for each language group. For the sample as a whole, SWAN total score and the inattention subscale have good inter-rater reliability, while the reliability for the hyperactivity subscale was fair. The two groups obtained similar ICC estimates for the SWAN total score as well as for the inattention and hyperactivity subscales; the overlapping 95% confidence intervals indicate that inter-rater reliability estimates for the two language groups do not differ significantly from each other. Thus we found no evidence that children’s language ability significantly affected inter-rater agreement on either of the two SWAN subscales or the SWAN total score (see Fig. 2).

**Table 2:** Indices of agreement between parent and teacher ratings on the SWAN

	ICC (95% CIs)			Cohen’s Kappa	
	SWAN total	SWAN inattention	SWAN hyperactivity	Kappa (z)	% agreement (random expected)
Full sample	.61 (.52-.69)	.60 (.50-.69)	.51 (.41-.62)	.23 (3.96**)	82.00 (76.54)
LL group	.62 (.50-.73)	.50 (.36-.65)	.58 (.44-.71)	.16 (2.07*)	69.81 (63.87)
TL group	.38 (.22-.56)	.44 (.29-.61)	.32 (.16-.52)	.32 (.24**)	95.74 (93.73)

*Note*: * *p* < 0.05,** *p* < 0.001. ICC reliability coefficients can be interpreted as follows: <.40 are poor, .40-.59 fair, .60-.74 good and .75-1.00 excellent [47]. Cohen’s Kappa can be interpreted as follows: < 0 no agreement, .0–.20 poor, 0.21–0.40 fair, 0.41–0.60 moderate, 0.61–0.80 substantial and 0.81–1 almost perfect agreement [48]

### Does children’s language ability affect agreement between parents and teachers regarding potential ADHD case identification?

Table 3 displays rates of potential ADHD ‘caseness’ identification by parents and teachers for the whole sample and by language group (a breakdown by ADHD subtype is reported in Additional file 1: Table S3). Across the sample, parents identified 13/200 (6.50%) children as having significant symptoms of ADHD, while teacher’s identified three times as many children, 39/200 (19.50%); with the majority of additional cases being identified as a result of teachers endorsing more symptoms of inattention (see Additional file 1: Table S3). Cohen’s kappa indicates that parents and teacher have fair agreement regarding ADHD case identification [48] (see Table 2). However, parents and teachers only agreed on 8/44 (18.18%) potential ADHD ‘cases’, whereas agreement on non-ADHD cases was much higher, 156/192 (81.25%).

**Table 3:** Rates of potential ADHD identification by parents vs. teach

	Teacher								
	Whole sample			Low language			Typical language		
Parent	No ADHD	ADHD	Total	No ADHD	ADHD	Total	No ADHD	ADHD	Total
No ADHD	156	31	187	67	27	94	89	4	93
ADHD	5	8	13	5	7	12	0	1	1
Total	161	39	200	72	34	106	89	5	94

For children with LL, parents identified 12/106 (11.32%) children as potential ADHD cases. Again, teacher’s identified almost three times as many children with LL, 34/106 (32.08%). Rates of identification among children with TL were much lower for both parents and teachers; 1/94 (1.06%) versus 5/94 (5.32%) respectively, although teachers again identified many more cases than parents. Cohen’s Kappa indicates that parents and teachers have poor agreement regarding potential ADHD “caseness” for children with LL and fair agreement for children with TL [48] (see Table 2). Once again, agreement on potential ‘cases’ was much lower, 7/39 (17.95%) and 1/5 (20.00%) for LL and TL groups respectively, relative to agreement on non-cases, 67/99 (67.68%) and 89/93 (95.70%) for the LL and TL groups respectively.

## Discussion

The current study examined for the first time the extent to which children’s language ability affected the correspondence between parent and teacher ratings of inattention and hyperactivity/impulsivity using the SWAN in a large, representative sample of young children. The current study did not aim to evaluate the diagnostic accuracy of the SWAN but rather consider how different informants rate symptoms of ADHD in this population of children with varying language skill. A key finding is that children’s language competence significantly affected the magnitude of the discrepancy between parent and teacher ratings of ADHD symptomatology and the agreement between informants regarding potential ADHD case identification; with teachers being more likely to endorse symptoms of inattention particularly within the LL group.

### Correspondence between parent and teacher ratings on the SWAN

Consistent with previous research [11, 23, 24] parent and teacher ratings on the SWAN were moderately correlated and teachers tended to rate children as having more ADHD related weaknesses, particularly in terms of inattention, relative to parents. The inter-rater reliability for the SWAN and its subscales, as measured by ICCs, was generally good. Agreement regarding ADHD “caseness” was mixed; while there was good agreement about children who did not exhibit clinically significant symptoms of ADHD, teachers identified three times as many potential ADHD cases as parents. These data suggest adequate inter-rater reliability of the SWAN, yet highlight potential sources of disagreement between raters that will be important for researchers and practitioners who are using this dimensional measure of ADHD symptomatology to consider.

### Does children’s language ability moderate correspondence between parent and teacher ratings?

Children with LL were rated as having greater inattention and more hyperactivity than peers with TL by both parents and teachers [14, 49]. Moreover, teachers rated children with LL as having significantly greater ADHD symptoms than parents, particularly in the inattention domain. The inter-rater reliability, as measured by ICC, did not however differ between language groups although case agreement, as measured by Cohen’s kappa, was influenced by language status; with greater agreement between informants when rating children in the TL group (where fewer potential cases were identified) compared to the LL group (where more children were identified as potential cases).

The poorer agreement in identification of ADHD ‘caseness’ for children with LL may appear at odds with previous findings that agreement between parents and teachers is often higher in clinical versus non-clinical samples [20, 50]. However, clinical samples are subject to Berkson’s bias and the children within our LL group were identified as having low language from a community sample. Thus parents and teachers in the current study have likely spent less time discussing the child’s progress, strengths and weaknesses, which in clinical samples may result in a consensus view of the child’s behavioural profile. An important caveat is the low return rate of questionnaires from both parents and teachers (overall 57% of parents and 65% of teachers returned completed SWAN questionnaires). Children without SWAN questionnaires from both parents and teachers had significantly greater language, behaviour and early academic weaknesses relative to those in the current sample (Additional file 1: Table S1). It is therefore possible that agreement would be enhanced for these children with more severe language and behavioural deficits. Furthermore, given there were low numbers of ADHD ‘cases’, particularly in the TL group, it is possible that with a bigger sample of children which includes more ‘cases’ of ADHD, parent-teacher case agreement would be poorer.

There are several possible reasons why language ability may moderate the discrepancy between parent and teacher ratings and their agreement regarding caseness. If a child has difficulties in one developmental domain, such as language, respondents may be more sensitive to any difficulties the child might experience in other areas, such as attention and behaviour (an example of the “halo effect”). However, parents and teachers may be differentially susceptible to this negative halo effect given the differing demands of the environments in which they observe the child’s behaviour. Alternatively, given the different environments in which parents and teachers observe child’s behaviour, these informants may use different sources of information or reflect on different situations when rating a child’s behaviour. This might be particularly evident on questions tapping overlapping symptoms of ADHD and language difficulties [16]. Thus weaknesses on items such as remembering daily activities, listening to and following through on instructions may actually reflect a child’s language competence rather than their attention skills. It is also possible that language weaknesses negatively impact attention skills, and this association may be more noticeable in demanding environments such as the classroom particularly if children do not have appropriate support. All of these factors would accentuate discrepancy between parent and teacher ratings and reduce agreement regarding ADHD caseness.

### Clinical implications

While our study did not aim to evaluate the diagnostic accuracy of the SWAN, understanding the factors which contribute to both discrepancies and better agreement in parent and teacher ratings of ADHD symptomatology has important implications for assessment and diagnosis of ADHD. Child language is clearly an important factor in understanding these discrepancies.

Our findings suggest two further possibilities: first, inattention may be more apparent to teachers in children with lower language proficiency as these children are struggling to meet the academic and social demands of the classroom. Similarly, symptoms of hyperactivity/impulsivity may be less apparent within a structured classroom environment if children have strong language ability. Second, parents may be underreporting ADHD symptoms and instead attributing attention/behaviour problems to their child’s language difficulties (e.g. diagnostic overshadowing). At present, the developmental trajectories of children with discrepant parent-teacher ratings remain unclear, though children with language impairments are at high-risk for future attention and behavioural deficits [51, 52].

Our findings suggest that rates of co-occurring language and attention difficulties may be higher if teacher, rather than parent, ratings of ADHD symptomology are used to classify children. Rater discrepancies highlight the need to examine children within both academic and non-academic settings. Moreover, assessing the language ability of children presenting to clinicians with high symptoms of inattention at school may help to ensure that language difficulties are not missed and that an appropriate treatment plan is put into place.

It has been suggested that removing items reflecting language competence from ADHD scales improves the ability of scales to differentiate between cases of ADHD and language impairment [53], though it is currently unclear whether this is improvement is limited to parent ratings (which appear to be less sensitive to child language ability than teacher ratings). Furthermore, these items may highlight important functional impacts of ADHD and age appropriate language skill may serve as a protective factor in the expression of ADHD in the classroom. Thus further research elucidating the sources of co-morbidity in these two common developmental conditions is warranted. The involvement of Speech and Language therapists or other clinicians in the behavioural evaluation and observation of children with ADHD may also help to ensure that symptoms of language impairment are not mistaken for symptoms of attention deficit and vice-versa; guarding against both halo effects and diagnostic overshadowing. Indeed, providing children with language deficits appropriate support within the classroom may help them to attend despite their language limitations and reduce the likelihood of secondary behavioural difficulties. Furthermore, measuring the impact of language interventions on improving attention would further elucidate the causal relationships between language and attention.

## Conclusion

Although children’s language competence does not directly affect the inter-rater reliability of the SWAN as measured by ICC, it does moderate the discrepancy between parent and teacher ratings (particularly regarding inattention), and agreement between these informants regarding the identification of potential ADHD cases. Consideration of a child’s language ability is a crucial component to the diagnosis and treatment of children with potential ADHD.

## Additional file

Additional file 1: Recruitment flow diagram. **Table S1.** Differences between children included in the current sample (i.e. those with SWAN questionnaire data available from both parents and teacher) and those who are not included in the current sample. **Table S2.** Mean (SD) parent and teacher ratings on the SWAN for the whole sample and by language group, **Figure S2.** Marginal means of SWAN total score as rated by parent (dark grey bars) and teachers (light grey bars) for typical language (TL) and low language (LL) groups; error bars are 95% CIs. Description of findings shown in **Figure S2.** **Table S3.** rates of potential ADHD subtype identification by parents vs. teachers for the whole sample and by language group. (DOCX 95 kb)

## Abbreviations

ADHD: Attention-deficit/hyperactivity disorder
CCC-S: Children’s communication checklist-short
CI: Confidence interval
EYFSP: Early years foundation stage profile
ICC: Inter-class correlation
LL: Low language
ODD: Oppositional defiant disorder
SCALES: Surrey communication and language in education study
SDQ: Strengths and difficulties questionnaire
SWAN: Strengths and weaknesses of ADHD symptoms and normal behaviour
TL: Typical language

## Footnotes

1 There were no differences between children who did and did not have both parent and teacher SWAN questionnaires returned with regard to age, percent males, percent with abnormal ratings on the SDQ, percent with known medical diagnoses or percent not achieving a ‘good level of development’ on the EYFSP. The 200 children in the current sample, however, were from less deprived neighbourhoods (Income Deprivation Affecting Children Index postcode rankings [54]) and had better overall teacher ratings for language and communication skills (CCC-S), behaviour (SDQ) and early educational attainment (EYFSP) in Reception, and better language composite scores in Year 1 than those who did not have both parent and teacher SWAN questionnaires returned. In addition, fewer children in the current sample met our criteria for low language (based on their CCC-S) scores than those not in the current sample (see Additional file 1: Table S1).
