# Supplementary material for: Does a child’s language ability affect the correspondence between parent and teacher ratings of ADHD symptoms?
Source: BMC Psychiatry. 2017 Apr 5;17:129. doi: 10.1186/s12888-017-1300-8 (PMC5382365; doi:10.1186/s12888-017-1300-8)
Supplement: Additional file 1: — Recruitment flow diagram. Table S1. Differences between children included in the current sample (i.e. those with SWAN questionnaire data available from both parents and teacher) and those who are not included in the current sample. Table S2. Mean (SD) parent and teacher ratings on the SWAN for the whole sample and by language group, Figure S2. Marginal means of SWAN total score as rated by parent (dark grey bars) and teachers (light grey bars) for typical language (TL) and low language (LL) groups; error bars are 95% CIs. Description of findings shown in Figure S2. Table S3. rates of potential ADHD subtype identification by parents vs. teachers for the whole sample and by language group. (DOCX 95 kb) [file 12888_2017_1300_MOESM1_ESM.docx]

Supplementary material

Figure S1 recruitment flow diagram


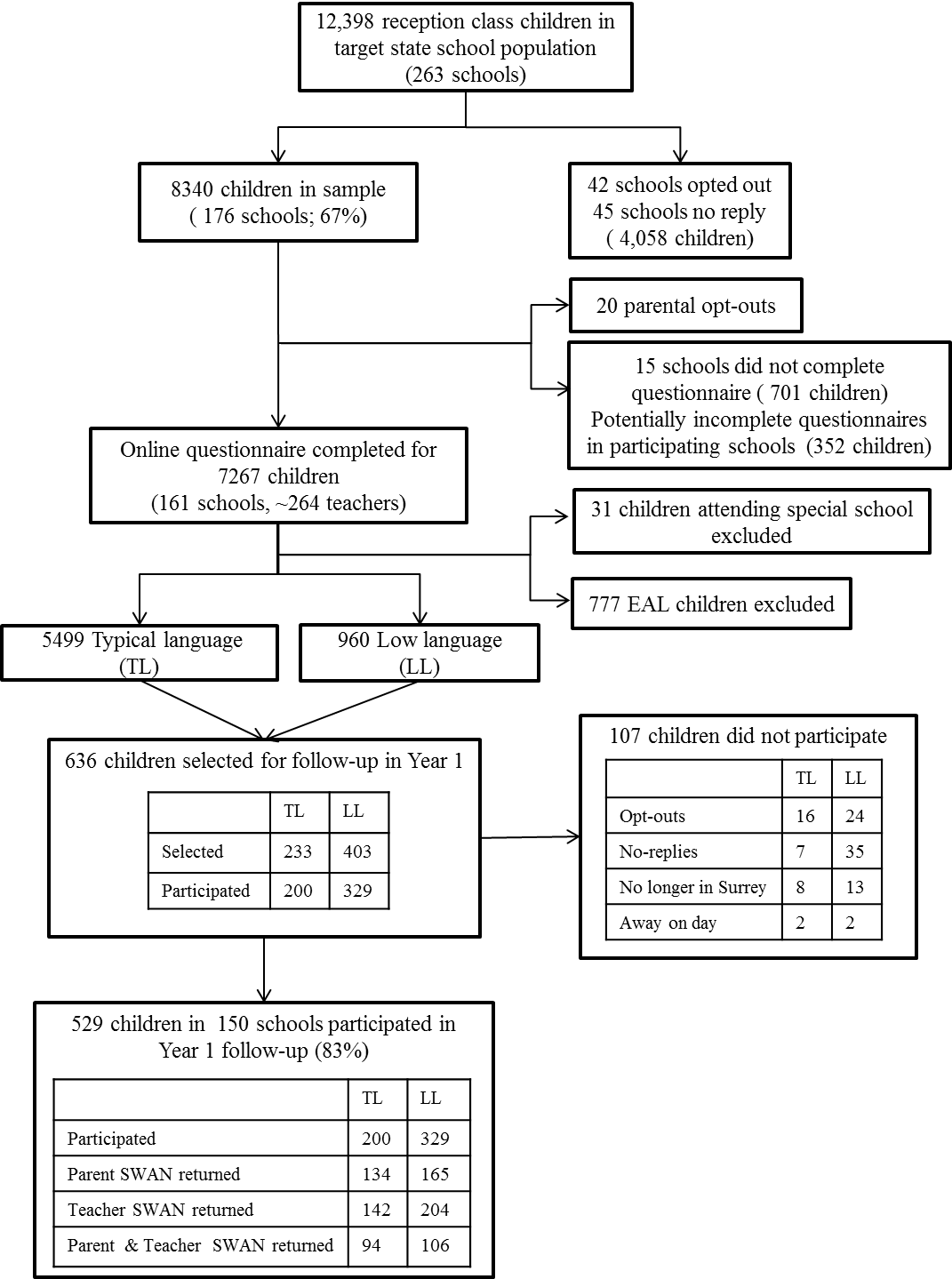


Table S1 Differences between children included in the current sample (i.e. those with SWAN questionnaire data available from both parents and teacher) and those who are not included in the current sample

|  | Current sample | Not in Current sample | F Values | *p* Values |
| --- | --- | --- | --- | --- |
| N | 200 | 329 |  |  |
| Age | 71.88 (4.72) | 71.48 (4.71) | F(1,528)=0.88 | p=.35 |
| % male | 49.00 | 54.71 | Chi2(1)=1.63 | p=.20 |
| SES (IDACI postcode)^1^ | 22871.81 (7590.24) | 20447.95 (7724.16) | F(1,528)=12.52 | p<.001 |
| CCC-S (raw score/max 39) | 15.62 (12.02) | 18.72 (11.06) | F(1,528)=8.78 | p<.01 |
| CCC-S (z-score) | .59 (1.17) | .92(.96) | F(1,528)=11.05 | p<.001 |
| % low language (CCC-S) | 53.00 | 67.78 | Chi2(1)=11.56 | p<.01 |
| SDQ total | 7.85 (6.21) | 9.03 (6.75) | F(1,528)=4.26 | p<.05 |
| % abnormal behaviour (SDQ) | 14.00 | 17.33 | Chi2(1)=1.02 | p=.31 |
| % known diagnosis | 9.00 | 6.69 | Chi2(1)=0.95 | p=.33 |
| EYFSP total | 32.07 (9.25) | 30.26 (8.16) | F(1,528)=5.18 | p<.05 |
| % not achieving GLD | 60.50 | 68.39 | Chi2(1)=3.42 | p=.06 |
| Language composite (z-score)^2^ | -.23 (1.13) | -.71 (.99) | F(1,505)=23.24 | p<.001 |

*Note: ^1^Income Deprivation Affecting Children Index (IDACI) rank scores were obtained from home post-codes and provided a measure of neighbourhood deprivation, reflecting family receipt of means tested benefits.^48^ IDACI scores in this sample ranged from 4686 (most deprived) to 32471 (most affluent); ^2^Data only available for those who completed all 6 core language tasks: N in current sample = 192, N not in current sample = 314.*

Table S2 mean (SD) parent and teacher ratings on the SWAN for the whole sample and by language group

|  | N | Parent | Teacher |
| --- | --- | --- | --- |
| *Whole sample* |  |  |  |
| SWAN Inattention | 200 | 38.51 (9.45) | 35.66 (13.31) |
| SWAN Hyperactivity | 200 | 38.64 (9.45) | 39.04 (12.23) |
| SWAN total | 200 | 77.15 (17.95) | 74.69 (24.50) |
| *Low language (LL)* |  |  |  |
| SWAN Inattention | 106 | 34.87 (9.03) | 28.86 (11.80) |
| SWAN Hyperactivity | 106 | 36.26 (9.42) | 34.10 (11.23) |
| SWAN total | 106 | 71.13 (17.50) | 62.96 (21.65) |
| *Typical language (TL)* |  |  |  |
| SWAN Inattention | 94 | 42.62 (8.18) | 43.32 (10.47) |
| SWAN Hyperactivity | 94 | 41.31 (8.80) | 44.60 (10.90) |
| SWAN total | 94 | 83.93 (15.99) | 87.91 (20.55) |

Figure S2: Marginal means of SWAN total score as rated by parent (dark grey bars) and teachers (light grey bars) for typical language (TL) and low language (LL) groups; error bars are 95% CIs.

Figure S2 shows that overall, there was a significant effect of language group indicating that children with LL were rated as exhibiting more ADHD symptoms than children with TL (SWAN total score: z=-7.91, p<.001). The effect of rater was not significant (SWAN total score: z=-1.64, p=.10). However, there was also a significant respondent x language group interaction (SWAN total score: z=-4.77, p<.001) indicating that the difference between parent and teacher ratings on the SWAN total score was greater for children with LL than for children with TL. Simple effects revealed that although both parents and teachers rated children with LL as having increased attention/behaviour deficits relative to TL peers (z=-4.73, p<0.001 and z=-9.22, p<0.001 respectively), teacher ratings of the LL group were significantly worse than parent ratings (z=-4.67, p<.001). In contrast, teacher ratings of the TL group were better than parents (z=2.15, p<.05), thought this effect was somewhat smaller as evident by overlapping CIs.

Table S3 rates of potential ADHD subtype identification by parents vs. teachers for the whole sample and by language group.

|  | Teacher | | | | | | | | | | | | | | |
| --- | --- | --- | --- | --- | --- | --- | --- | --- | --- | --- | --- | --- | --- | --- | --- |
|  | Whole sample | | | | | LL | | | | | TL | | | | |
| Parent | No ADHD | I | H/I | C | Total | No ADHD | I | H/I | C | Total | No ADHD | I | H/I | C | Total |
| No ADHD | 156 | 23 | 3 | 5 | 187 | 67 | 21 | 1 | 5 | 94 | 89 | 2 | 2 | 0 | 93 |
| ADHD type |  |  |  |  |  |  |  |  |  |  |  |  |  |  |  |
| Inattentive (I)^a^ | 3 | 1 | 0 | 0 | 4 | 3 | 1 | 0 | 0 | 4 | 0 | 0 | 0 | 0 | 0 |
| Hyperactive/Impulsive (H/I)^b^ | 1 | 0 | 0 | 2 | 3 | 1 | 0 | 0 | 2 | 3 | 0 | 0 | 0 | 0 | 0 |
| Combined (C)^c^ | 1 | 0 | 0 | 5 | 6 | 1 | 0 | 0 | 4 | 5 | 0 | 0 | 0 | 1 | 1 |
| Total | 161 | 24 | 3 | 12 | 200 | 72 | 22 | 1 | 11 | 106 | 89 | 2 | 2 | 1 | 94 |

Notes: ^a^ ≥ 6 I symptoms and <6 H/I symptoms; ^b^ ≥ 6 H/I symptoms and <6 I symptoms; ^c^ ≥ 6 I symptoms and ≥ 6 H/I symptoms
